# Fibrin Glues: Proteins, Mechanism of Action, Classification, and Application

**DOI:** 10.3390/ijms27010447

**Published:** 2025-12-31

**Authors:** Ekaterina A. Levicheva, Daria D. Linkova, Ekaterina A. Farafontova, Yulia P. Rubtsova, Irina N. Charykova, Diana Ya. Aleynik, Marfa N. Egorikhina

**Affiliations:** Federal State Budgetary Educational Institution of Higher Education, Privolzhsky Research Medical University of the Ministry of Health of the Russian Federation, 603005 Nizhny Novgorod, Russia; kate.lekat@yandex.ru (E.A.L.); linckovadaria@yandex.ru (D.D.L.); ekaterina_farafontova@mail.ru (E.A.F.); rubincherry@yandex.ru (Y.P.R.); irina-ch0709@yandex.ru (I.N.C.); daleynik@yandex.ru (D.Y.A.)

**Keywords:** fibrin glue, fibrinogen, thrombin, hemostasis, bleeding, surgery

## Abstract

This research paper is concerned with fibrin glues, used as effective tools to stop bleeding in the case of wounds and surgical interventions. The paper provides data on the various fibrin glues—both currently used in practice and being developed, their sources, mechanisms of action, and properties. Such glues are biocompatible and are characterized by good adhesive and hemostatic properties. These characteristics mean that fibrin glues are currently widely used across a range of surgical applications. Such glues can be used independently or in combination with mechanical techniques, being particularly suitable for difficult-to-access parts of the body. The combined use of fibrin glues with various biologically active substances (BASs)—such as antibiotics and growth factors—and with cell therapy is a promising approach. Such adjuncts enhance the effectiveness of the glues and help to optimize the therapies. This research paper presents the latest data from studies using various experimental models demonstrating the increased efficacy of fibrin glues used in combination with BASs. We also report on the ongoing development of new fibrin glues for long-term use and with optimized formulations. Studies on the interactions of these glues with cells and tissues are supporting the creation of a new generation of fibrin glues with adjustable properties.

## 1. Introduction

Local hemostasis in surgical interventions and in the case of major injuries is still a significant issue in surgery [1,2,3]. Effective control of hemostasis during interventions is critical not only to ensuring the best possible view of the surgical site but also to preventing complications related to blood loss in the postoperative period [4]. Parenchymal bleeding is particularly difficult to control [5]. Globally, postoperative hidden bleeding is a cause of high mortality and disability [6,7,8]. Furthermore, there are clinical situations when the use of mechanical tissue adhesion techniques (surgical sutures or staples) is problematic, for example, in ophthalmic surgery [9,10]. Moreover, the growing demand for control of hemostasis and for means of tissue sealing is promoting both the development of new biological glues and improvements in existing sealing materials [11]. The introduction of these new glues into clinical practice ensures successful control of hemostasis, maximizing wound sealing, especially in difficult-to-reach areas, as well as accelerating healing and tissue regeneration [12].

**Figure 1:**
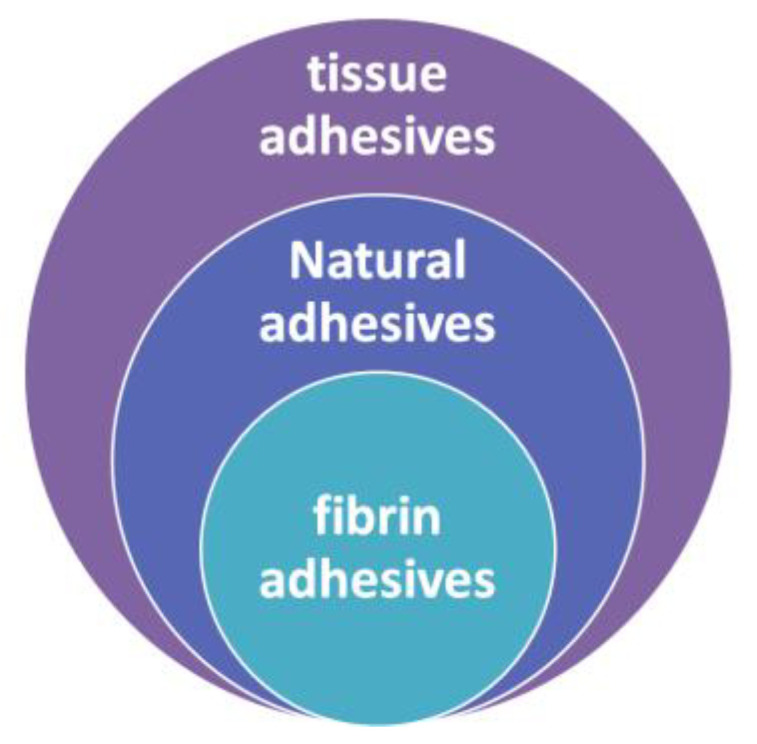
FGs in the classification of biological glues (the diagram is based on the findings of Mazur M. et al. [14]).

Depending on their composition, biological glues can be divided into synthetic and natural types [13,14]. Fibrin glues (FGs) hold a special place among the natural biological glues (Figure 1).

Fibrin glues (FGs), also known as fibrin sealants (FSs) or fibrin adhesives (FAs), represent a type of biological glue for topical application; they are obtained by mixing solutions of fibrinogen and thrombin to form fibrin clots that simulate the final stages of the blood coagulation cascade [15,16].

Fibrin glues are already widely used due to their advantageous properties, such as in situ polymerization, high adhesion, biocompatibility, biodegradability, and biological activity. They are in high demand in almost all areas of surgery: general surgery, neurosurgery, vascular and nephron-sparing surgery, traumatology and orthopedics, ophthalmic surgery, and reconstructive plastic surgery. In accordance with their purpose for covering tissue defects, these fibrin sealants are most often selected to stop bleeding and hermetically to seal wound surfaces of various organs and tissues (in addition to, or instead of, surgical sutures) [17,18].

As noted above, the principle of action of FGs is based on simulation of the blood clotting coagulation cascade with the conversion of fibrinogen to fibrin being influenced by thrombin. This approach determines the glues’ high tissue adhesion. Furthermore, unlike synthetic sealants, fibrin glues are biodegradable and biologically resorptive, forming products that are completely utilized by the human body. This allows them to be used for hidden hemostasis without the need for repeated interventions for removal of the adhesive material [13].

The need for effective hemostasis and sealing materials, the increasing number of surgical interventions, and the growing demand for minimally invasive surgical techniques have ensured the growth of the global market for fibrin glues. Such hemostatic materials now represent about 60% of the market, as these glues are essential for hemostasis in complex surgical interventions. The market is expected to reach USD 46,305 million in 2025 and USD 934.68 million by 2029. North America and Europe dominate the market, while Asia Pacific shows a significant growth potential. Growing healthcare infrastructure and increasing numbers of surgical interventions in emerging markets continue to provide opportunities for further market growth. Ongoing research and development aimed at creating new fibrin glue formulations, including those with extended shelf lives and improved performance, also add up to growth opportunities. The collaboration of medical device manufacturers, research institutes, and healthcare providers will facilitate the development and commercialization of innovative products based on fibrin glues. However, there are several factors that constrain the growth of their global market. For example, the high cost of human-origin components for fibrin glues may limit their general use, especially in regions with limited healthcare budgets. Strict regulatory procedures for medical glues can delay product market authorization and thus slow down market growth. However, technological developments and the expansion of fibrin glue applications, such as improved delivery systems and formulations, contribute to market expansion [19,20,21].

The purpose of our research is to summarize data on current fibrin glues, their potential applications, and areas for improvement.

## 2. Variety of Fibrin Glues

At present, a great variety of fibrin glues are available on the global market. Table 1 summarizes information on the major fibrin glues available internationally. All of them share common characteristics: they are supplied as two-component systems containing the main active ingredients fibrinogen and thrombin. In addition to these active ingredients, the products often include dual-syringe delivery and mixing systems. Based on the degree of ‘foreignness’ of the main components to a patient, these fibrin sealants can be classified into xenogeneic, allogeneic, or autologous types.

Xenogeneic fibrin glues use fibrinogen of animal origin (for example, made from pig or bovine blood) as the main component. The best-known xenogeneic fibrin glues are Hemaseel™ (Heamacure Corp., Montreal, Canada) and BIOSEAL (Guangzhou Bioseal Biotechnology Co., Ltd., Gaopu Road, Tianhe District, China). The advantages of xenogeneic FGs include the availability of large quantities of primary raw materials, allowing for a significant yield of the finished product and therefore low overall cost. However, xenogeneic materials also have natural disadvantages. First of all, there is a high risk of the transfer of transmissible infections and of immunological reactions in patients, particularly pyrogenicity, during bioresorption.

Unlike the other fibrinogen glues shown in Table 1, user guides for xenogeneic sealants are not publicly available. Thus, it has not been possible to obtain detailed information on the compositions of such fibrinogen glues and their properties. However, partial information on the composition and manufacturing technique used for Hemaseel™ is briefly described in the abstract of an article in *Surgical Adhesives & Sealants* [22]. An in vitro study showed that intensive dry heat treatment, to which the fibrinogen component of the material is subjected for viral inactivation, does not critically affect the conformation of the fibrinogen molecule [23]. The use of Hemaseel™ had been demonstrated in preclinical studies. For example, studies on rats proved that Hemaseel™ prevented the formation of intraperitoneal adhesions more effectively than did a cryoprecipitate-based material [24]. A positive effect of Hemaseel™ on the engraftment of cultured epidermal grafts was demonstrated in an athymic mouse model [25]. However, no information was found on its clinical use. No open access publications were found on BIOSEAL either, although several clinical studies without description of their results are available on the Good Clinical Practice Network website [26,27,28,29].

In the case of autologous FGs, the fibrinogen, or both the fibrinogen and thrombin, is obtained from the patient’s own blood. The blood is typically processed in a closed-circuit automated system that produces syringes containing the finished product. The preparation process can proceed at the patient’s bedside. This approach has the advantage of high safety and biocompatibility due to the autologous nature of the components. However, this very autologous nature also has several disadvantages. For example, autologous medications are difficult to standardize, as each patient’s blood parameters are individual. Variability in the final concentrations of the main components of the fibrin glue directly impacts its final properties, such as polymerization time and adhesive characteristics. Even when the system manufacturer standardizes the components on the basis of their fibrinogen and thrombin concentrations, the problem of variability in the final product volume persists, sometimes making it impossible to obtain a sufficient quantity of the material. Moreover, the use of closed-loop systems largely determines the high cost of the finished product. Studies aimed at overcoming these disadvantages are driving the development of new systems to produce autologous fibrin sealants [30,31].

Allogeneic fibrin sealants are the most common and in demand. In these materials, the fibrinogen and thrombin components can be supplied either lyophilized and packaged with appropriate solvents or as frozen solutions in pre-filled syringes ready for use immediately after thawing. Typically, both components are produced from donor blood pools, enabling increased standardization of the final product, both in terms of the concentrations of the active ingredients and the volume of the finished material. Despite complex purification steps and strict quality control, some researchers note that a minimal risk of infectious disease transmission remains with individual lots [30]. Currently, there are more than 10 types of allogeneic fibrin sealant on the market, the best known of which are shown in Table 1. The most popular allogeneic materials for clinical practice are the Tissucol^®^ Kit (Baxter, AG, Vienna, Austria), TISSEEL Kit (Baxter Healthcare Corporation, Deerfield, Illinois, USA), and Evicel^®^ (Omrix Biopharmaceuticals Ltd., Nes Ziyyona, Israel). Due to the high demand for FGs in clinical practice, the development of new allogeneic FGs is ongoing, as is the search for ways to reduce the cost of their production and, thus, increase their availability. Moreover, there is a need to update the composition of current FGs to add specific properties (for example, antibacterial activity and increased affinity for bone tissue).

**Table 1:** Specifications of commercially available fibrin glues.

Name	Manufacturer	Pharmaceutical Form and Presentation	Active Constituents	Foreignness of Major Components	Polymerisation Time	Storage Conditions	Shelf Life
Hemaseel™ [22]	Heamacure Corp., Westmount, Canada	Consists of two ingredients: a clotting protein containing mainly fibrinogen, and a thrombin component.	No. 1 is a clotting protein from bovine blood; No. 2 is a processed commercial thrombin medication.	Xeno-	No public data	No public data	No public data
BIOSEAL [29]	Ethicon, Inc., Guangzhou Bioseal Biotechnology Co., Ltd., Gaopu Road, Tianhe District, China	Fibrin sealant based on porcine plasma, consisting of thrombin and fibrinogen.	No. 1 is fibrinogen from porcine plasma; No. 2 is thrombin from porcine plasma.	Xeno-	No public data	No public data	No public data
Vivostat^®^ [32]	Vivostat A/S, Alleroed, Denmark	A fully automated, closed system for fibrin glue preparation from the patient’s blood.	No. 1 is a fibrin solution from the patient’s blood plasma; No. 2 is a buffer solution (*manufacturer does not disclose the composition*).	Auto-	2 min	From −18 °C to −25 °C	Up to 5 weeks
CRYOSEAL^®^ FS SYSTEM [33]	ThermoGenesis Corp., Rancho Cordova, CA, USA	Medical device that simultaneously produces cryoprecipitate and thrombin from a sample of autologous plasma. When the components are mixed, they form fibrin sealant.	No. 1 is a cryoprecipitate from the patient’s blood plasma; No. 2 is thrombin from the patient’s blood plasma.	Auto-	3–5 min	From +34 °C to +37 °C	Up to 6 h
VISTASEAL™ [34]	Ethicon, Inc., INSTITUTO GRIFOLS, S.A., Barcelona, Spain	Frozen, pre-filled syringes with components No. 1 and No. 2, combined in a single-use carrier.	No. 1 is a sterile solution with pH 6.5–8.0. Active ingredient: human fibrinogen (80 mg/mL). Auxiliary ingredients: sodium citrate, sodium chloride, arginine, L-isoleucine, L-glutamic acid, monosodium, and water for injection. No. 2 is a sterile solution with pH 6.0–8.0. Active ingredient: human thrombin (500 IU/mL). Auxiliary ingredients: calcium chloride, human albumin, sodium chloride, glycine, and water for injection.	Allo-	2–4 min	−18 °C or colder	Up to 2 years
Evicel ^®^ [35]	Omrix Biopharmaceuticals Ltd., Nes Ziyyona, Israel	Package containing vials of frozen components No. 1 and No. 2. Modular application device.	No. 1 is a sterile solution, pH 6.7–7.2. Active ingredient: human fibrinogen concentrate (55–85 mg/mL). Auxiliary ingredients: arginine hydrochloride, glycine, sodium chloride, sodium citrate, calcium chloride, water for injection; No. 2 is a sterile solution, pH 6.8–7.2. Active ingredient: human thrombin (800–1200 IU/mL). Auxiliary ingredients: calcium chloride, human albumin, mannitol, sodium acetate, water for injection.	Allo-	4–6 min	−18 °C or colder	Up to 2 years
Tissucol^®^ Kit [36]	Baxter, AG, Vienna, Austria	Set of lyophilised components No. 1 and No. 2, solvents for the components, Duploject spraying system for fibrin glue application.	No. 1 is lyophilized Tissucol powder and aprotinin solution as a solvent; No. 2 is lyophilized thrombin of various concentrations (4 IU and 500 IU) and calcium chloride solution as a solvent	Allo-	40–60 s	From +2 °C to +8 °C	2.5 years
TISSEEL Kit [37]	Baxter Inc., Deerfield, Illinois, USA	Frozen, pre-filled syringes with components No. 1 and No. 2, complete with the Duploject spraying system.	No. 1—Active ingredients: fibrinogen (72 mg/mL), factor XIII (1.2 IU/mL), aprotinin (3000 KIU/mL). Auxiliary ingredients: human albumin, trisodium citrate, histidine, niacinamide, polysorbate, water for injection; No. 2—Active ingredients: thrombin (400 IU/mL), calcium chloride (36 micromoles/mL). Auxiliary ingredients: human albumin, sodium chloride, water for injection.	Allo-	4–5 min	−20 °C or colder	2 years
BERIPLAST P COMBI-SET [38]	CSL Behring GmbH, Hattersheim am Main, Germany	Supplied as a powder and solvent. Administration kit: sterile disposable tuberculin syringes, Pantaject application kit, sprayers and cannulas.	Vial 1: Concentrate: fibrinogen 90 mg/mL, Auxiliary ingredients: factor XIII (60 IU/mL), human albumin, L-arginine, L-isoleucine, sodium chloride, sodium citrate dihydrate, sodium L-glutamate monohydrate. Vial 2: Solvent: bovine lung aprotinin 1000 KI (kallikrein inactivator unit), sodium chloride, water for injection. Vial 3: Concentrate: thrombin 500 IU/mL, sodium chloride, sodium citrate dihydrate. Vial 4: Solvent: calcium chloride dihydrate, water for injection.	Allo- Xeno-	Not specified	From +2 °C to +8 °C	2 years
KRIOFIT medical glue [39]	LLC “PLASMA-FTK”, Moscow, Russia	Set to prepare two-component fibrin-thrombin glue: a cassette of two syringes with a common plunger.	No. 1 is a donor plasma fraction containing concentrated fibrinogen; No. 2 is activated thrombin.	Allo-	Not specified	−18 °C or colder	1 year

## 3. Sources and Techniques for Obtaining the Key Fibrin Glue Components—Fibrinogen and Thrombin

### 3.1. Fibrinogen

The principal components of fibrin glues are fibrinogen and thrombin. Fibrinogen is a plasma protein that has a key role in hemostasis. It is a large glycoprotein with a molecular weight of 340 kDa. The fibrinogen molecule consists of two identical subunits, each composed of three non-identical polypeptide chains: Aα, Bβ, and γ. These chains are linked by 29 disulfide bonds and form several structural regions: two distal D regions, one central E region, and two αC regions [40]. Fibrinogen is the main constituent of fibrin glues, being the principal component involved in the blood clotting process and thus the main driver of hemostasis. Fibrinogen’s impact has also been noted in such cellular processes as proliferation, differentiation, adhesion, migration, healing, inflammation, and angiogenesis [41]. This additional range of activity ensues from fibrin glues not only acting as hemostatic agents, but also from their provision of a matrix that acts as a scaffold in the process of regeneration of areas of damage.

Fibrinogen can be obtained from human or animal blood plasma. Fibrin glues are manufactured using fibrinogen obtained from the plasma of one or more donors using various techniques. One of the first laboratory techniques to obtain fibrinogen was “chemical precipitation”. This method is still used today. Such chemical precipitation of fibrinogen from blood plasma is associated with changes in the protein’s solubility under various precipitating agents. For instance, ethanol precipitation is widely used for the selective isolation of fibrinogen [42]. Despite the fact that this technique has shown high efficiency in isolating fibrinogen with minor losses in concentration compared to the original plasma [43], it has several disadvantages. The use of this technique is directly associated with high risks of protein denaturation by the ethanol and, thus, a decrease in its functional activity, especially when the temperature rises above 0 °C. It has been demonstrated that the use of high ethanol concentrations (over 100 mL/L) may result in significant fibrinogen losses [42]. A further issue with this technique is the need for subsequent purification of the protein from the ethanol while preserving its functional activity. The impact of ethanol is less critical when the protein solution is frozen, due to a decrease in the molecular mobility of substances and the slower diffusion of ethanol and water molecules. This reduces the denaturing effect of the ethanol on the protein, thereby preserving its functional activity [44].

Another isolation technique is fibrinogen precipitation using ammonium sulfate. The advantages of this approach include its efficiency, allowing for the extraction of a high proportion (up to 70–80%) of the fibrinogen, and its relative simplicity. One of the main disadvantages is the need for further purification of the extract to remove residual ammonium sulfate [43]. Another technique based on the chemical precipitation of fibrinogen is protein precipitation with polyethylene glycol (PEG). As with the previously described techniques, it is characterized by high efficiency, but similarly requires subsequent purification of the fibrinogen [45].

A clear advantage of chemical precipitation is the ability to obtain high fibrinogen concentrations. However, the process of purifying the protein from the precipitating agent is often more labor-intensive than the precipitation itself. Inability to completely remove precipitating agents from the final product is also a significant issue. Here, it is difficult to find a balance between precipitation efficiency and the resulting purity of the final product. For example, raising the PEG concentration does significantly increase the yield of fibrinogen, but the number of impurities in the final product increases. Unfortuately, any residual chemical compounds can affect the physical and chemical properties of the fibrin glue. For example, residual ethanol can accelerate coagulation and activate factor XIII, making the fibrin clot less resistant to stretching. A further clear disadvantage of these techniques is the significant risk of bacterial contamination when chemical precipitation is conducted in industrial settings in open systems [46]. When the procedure is performed in this way, even the use of sterile disposable equipment does not reduce the high risk of such contamination [47].

Cryoprecipitation, which can be implemented in a closed circuit [48,49], is currently used as an alternative technique to reduce the risk of pathogen transmission. This technique is based on freezing the blood plasma followed by its slow thawing at a temperature of +1 to +6 °C, resulting in the formation of a precipitate—the cryoprecipitate. This cryoprecipitate contains cryoproteins such as coagulation factors VIII, XIII, von Willebrand factor and fibronectin, as well as fibrinogen [50]. The concentration of proteins in the cryoprecipitate is significantly higher than in the original blood plasma. This is an undoubted advantage, as the proteins in it provide additional hemostatic properties, can stabilize the fibrin clot, and enhance the regenerative potential of the fibrin matrix. Other advantages of cryoprecipitation are that the isolation process does not require additional potentially cytotoxic precipitating agents, and thus, additional protein purification cycles are not required. However, a significant disadvantage of this technique is the relatively low degree of fibrinogen extraction. Typically, it ranges from 24% to 36% of the original fibrinogen content of the blood plasma [51].

### 3.2. Thrombin

The second component essential for manufacturing fibrin glues is thrombin. Thrombin is a plasma enzyme that has a crucial role in the blood clotting process, catalyzing the conversion of fibrinogen to fibrin, which forms the structural basis of the blood clot. Thrombin is also known as coagulation factor IIa. It functions as a serine protease, activating other coagulation factors (V, VIII, XI, XIII) and promoting platelet activation and aggregation [52].

Thrombin is produced using a variety of techniques, including isolation from human or animal plasma, or by recombinant production. Thrombin isolation from plasma is typically carried out in several stages. In the first stage, prothrombin is isolated from plasma using cryoprecipitation, ion exchange chromatography (diethylaminoethyl, DEAE-IEX), or heparin affinity chromatography. In the second stage, DEAE-IEX and immobilized metal affinity chromatography (IMAC) are applied. After isolation, prothrombin is activated to thrombin, which is then purified, for example by hydrophobic interaction chromatography (HIC), and concentrated, typically by ultrafiltration [53].

In the case of recombinant production, the process involves expressing recombinant prothrombin in cell lines (for example, CHO cells). The human thrombin can then be obtained by activating the prothrombin, using a recombinant enzyme. This technique ensures high productivity while minimizing the risk of contamination with animal-derived substances [54].

In addition to the above, there are alternative techniques to produce thrombin. It can be generated by adding excess calcium ions to citrated plasma. This initiates thrombin production by activating the coagulation cascade. However, this technique is characterized by low thrombin yield and its rapid degradation due to inhibitory proteins in the plasma [55]. Another technique for thrombin production is based on the use of solid adsorbents followed by elution and activation of the resulting prothrombin [56].

## 4. Mechanisms of Interaction Between Fibrin Glue Components and the Potential for Further Modifications

**Figure 2:**
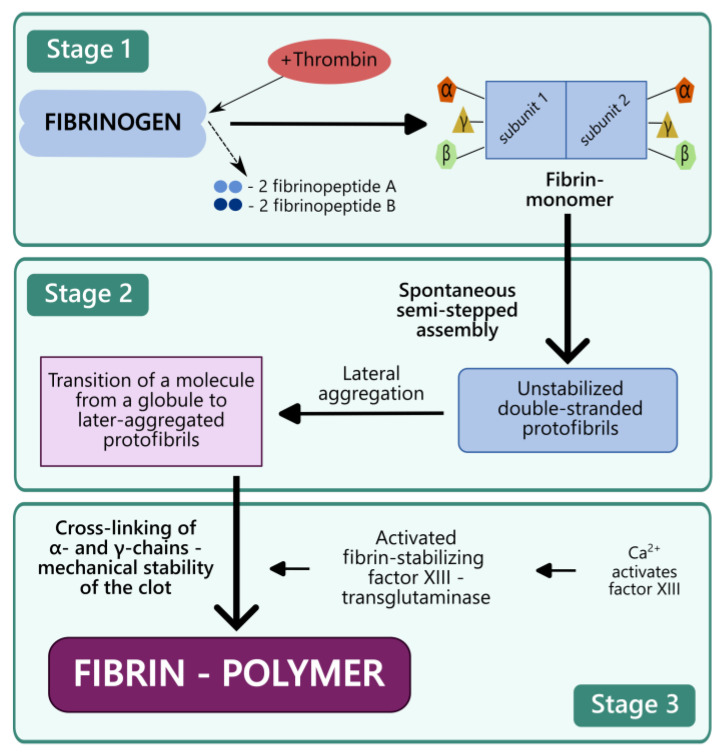
Mechanism of fibrin matrix formation.

The principle of action of fibrin glues is based on simulation of the coagulation cascade of blood clotting. The resulting cured glue is fibrin, a high-molecular-weight protein that has the form of smooth or striated fibers, the clumps of which would normally form the framework of the thrombus during blood clotting [57]. The fibrin is derived from fibrinogen, a plasma glycoprotein with a complex structure [58]. Fibrin formation occurs in several stages. In the first stage, under the impact of the enzyme thrombin, two A fibrinopeptides and two B fibrinopeptides are cleaved from the fibrinogen molecule (from the Aα and Bβ chains, respectively) (Figure 2). Fibrin monomers are formed; each consists of two identical subunits linked by disulfide bonds. The individual subunits contain three different polypeptide chains, called α, β, and γ [59].

In the second stage, fibrin monomers spontaneously assemble in a semi-stepwise pattern, forming unsterilized two-chain protofibrils. Lateral aggregation of the protofibrils occurs, representing the self-assembly of fibrin fibers. Lateral aggregation of unsterilized protofibrils includes the transition of the molecule from a globular state to a fibrillar state [60].

In the third stage, the laterally aggregated protofibrils are changed due to the enzymatic action of activated fibrin-stabilizing factor XIII—transglutaminase. It should be noted that no information on the use of transglutaminase as a component of any of the fibrin glues was found during our research. However, factor XIII is an important component for the formation of a natural fibrin clot and is certainly present in the fibrin glues made using plasma cryoprecipitate. The role of factor XIII in hemostasis is well studied. It ensures mechanical stability for the clot by cross-linking the α- and γ-chains of fibrin with covalent bonds and thus protects it from fibrinolytic degradation [61]. As a result, the fibrin aggregate is stabilized into fibrin polymer, ensuring the long-term stability and mechanical integrity of the fibrin clot [62]. Here, multiple lysine residues in the C-terminal domain of fibrin act as substrates for the transglutaminase [63]. The activated factor XIII uses a double substitution mechanism to cross-link the proteins. In the initial step, cysteine attacks the γ-carboxyamide group of glutamine (the glutamine acceptor), ejecting an ammonia molecule to form an intermediate thioester. In the second step, the reactive thioester is attacked by the ε-amino group of lysine (the amine donor), thereby displacing cysteine and forming a glutamine-lysine isopeptide bond. In the absence of lysine residues, water reacts with the intermediate thioester, converting glutamine to glutamic acid [64]. This has a negative effect on clot stability. Thus, lysine and glutamine are essential components of fibrin glues, providing excess lysine and glutamine residues for additional stabilization of the fibrin clot.

Divalent calcium ions also have a key role in fibrin clot formation. Calcium ions act as cofactors for the polymerization of fibrin monomers. Fibrinogen is known to have specific calcium binding sites, and the addition of Ca^2+^ to a fibrinogen solution significantly improves the mechanical properties of the clot. Calcium ions contribute to increased clot stability with respect to acid and thermal denaturation [65]. Ca^2+^ ions also induce fibrin formation from fibrinogen, increasing the reaction rate [66]. Moreover, calcium ions have a key role in the activation of fibrin-stabilizing factor XIII—transglutaminase [67]. Some of the commercially available fibrin glues contain calcium chloride as a source of divalent calcium ions, with it usually being included in the thrombin component of the glue. However, according to a number of publications, the use of fibrin glue that is lacking exogenous calcium chloride does not result in any negative side effects and maintains its clinical efficacy [51].

A widely used component of fibrin glues is isoleucine. Isoleucine (or its residues, to be more precise) has an important role in the activation and stabilization of TAFI (thrombin-activatable fibrinolysis inhibitor). The latter increases both the stability of the fibrin clot and its lifetime. TAFI is a procarboxypeptidase, which, through activation, can weaken the fibrinolysis of the fibrin clot [68]. TAFI is mainly synthesized in the liver [69] and circulates in blood plasma at a concentration of 4–15 μg/mL. However, in low concentrations (amounting to approximately 0.1% of the total), TAFI is also synthesized in platelets and is released upon platelet activation by thrombin. Despite the very low TAFI concentrations in platelets, it has been suggested that the TAFI platelet pool acts antifibrinolytically by locally increasing TAFIa activity due to the high concentration of platelets in the blood clot [70]. Although TAFI itself shows little intrinsic carboxypeptidase activity, its antifibrinolytic property depends on the carboxypeptidase activity of TAFIa [71]. TAFIa is formed by proteolytic cleavage of the arginine–alanine bond by trypsin-like proteases—thrombin or plasmin. This results in the release of the activation peptide from the catalytic part of TAFIa with its subsequent activation [69]. TAFIa inhibits fibrinolysis by disrupting plasmin-mediated feedback loops that promote a spike in plasmin formation. Plasmin can self-regulate in at least two ways. The first is based on the principle that continuous fibrin cleavage by plasmin creates new lysine residues in the C-terminal domain. They act as binding sites for plasminogen and tPA (tissue plasminogen activator).

tPA is a serine protease consisting of a single polypeptide chain that includes a signal peptide and a propeptide. tPA is present in the blood at low concentrations, unlike plasminogen, which circulates in the blood at a concentration of approximately 200 mg/L. However, tPA is stored in vascular endothelial cell vesicles and is actively released in the event of tissue damage. The enzymatic activity of tPA itself is quite low, but in the presence of fibrin, it can increase more than 100-fold. This increase is directly related to the formation of a ternary tPA-plasminogen-fibrin complex, through the formation of a bond between tPA and plasminogen with lysine residues in the C-terminal domain of fibrin. Under the influence of tPA, plasminogen is converted into its active form, the serine protease plasmin. Plasminogen is activated by peptide bond cleavage, resulting in the formation of a double-chain plasmin molecule consisting of a heavy and light chain. The light chain contains a proteolytically active site, whose interaction with fibrin leads to the formation of more lysine residues at the resulting newly formed C-terminal domains. Plasmin similarly interacts with single-chain tPA, converting it to a double-chain form. However, unlike plasminogen, single-chain tPA exhibits no less activity than its double-chain form [72,73]. Accordingly, it can be said that fibrin is not only a substrate but also a cofactor that triggers its own degradation. Several different mechanisms, in turn, are responsible for inhibiting the fibrinolytic system. tPA activity is suppressed by plasminogen activator inhibitors 1 and 2 (PAI). Plasmin itself is directly inhibited by α2-AP (α-2-antiplasmin) through the formation of the plasmin–antiplasmin (PAP) complex. When α2-AP binding capacity is exceeded by high plasmin concentrations, plasmin also begins to bind α2-macroglobulin. Furthermore, TAFIa cleaves lysines from the carboxyl C-terminus of any fibrin that has been partially degraded by plasmin, thereby reducing the binding of plasminogen and tPA to such fibrin and, consequently, decreasing plasmin formation [74]. Equally important is the process of cross-linking α2-AP with fibrin via activated factor XIII (FXIIIa), as a result of which α2-AP inhibits the formation of plasmin and the degradation of the fibrin clots [75].

The second mechanism is based on the ability of plasmin to independently generate further molecules of itself by proteolytically cleaving native Glu-plasminogen to create Lys-plasminogen. Lys-plasminogen is a more suitable substrate for tPA and does not have strict requirements for lysine residues in the C-terminal domain of partially degraded fibrin [76,77]. The decreased cofactor role of partially degraded fibrin in Lys-plasminogen activation is probably the reason for Lys-plasminogen activation being less susceptible to inhibition of the reaction by TAFIa. However, TAFI influences clot lysis via a threshold-dependent mechanism. Thus, as long as the TAFIa concentration remains above the threshold, it prevents the progression of the clot lysis chain reaction. The removal of accessible plasminogen binding sites on the C-terminal domain of fibrin, combined with the inhibition of Lys-plasminogen formation, are the basis of this phenomenon [78,79,80,81,82]. It is worth noting that α2-AP fibrinolysis inhibitors, especially in combination with FXIIIa, suppress fibrin clot degradation no less effectively than in the first pathway. The TAFIa prevents progress of the fibrinolytic chain reaction by directly inhibiting the conversion of Glu-plasminogen to Lys-plasminogen, inhibiting fibrin cofactor functions, and thus indirectly inhibiting the formation of plasmin from Glu-plasminogen [83].

Recent data indicate that TAFI influences clot lysis by means of a threshold-dependent mechanism. For instance, as long as the TAFIa concentrations remain above the threshold, TAFIa prevents the clot lysis chain reaction. The removal of accessible plasminogen binding sites at the C-terminal domain of fibrin, together with the inhibition of Lys-plasminogen formation, underlies this phenomenon. Once TAFIa concentrations fall below the threshold value, the plasminogen binding sites become accessible, plasmin is generated, and the number of lysine residues at the C-terminal domain increases exponentially using the plasmin-mediated feedback. Plasmin therefore becomes available to cut native Glu-plasminogen to form Lys-plasminogen, which is a better substrate for tPA, as its activation is not regulated [84]. Thus, any TAFIa concentration falling below the threshold results in an accelerated rate of lysis, and therefore termination of the TAFIa-mediated inhibition of such lysis.

**Figure 3:**
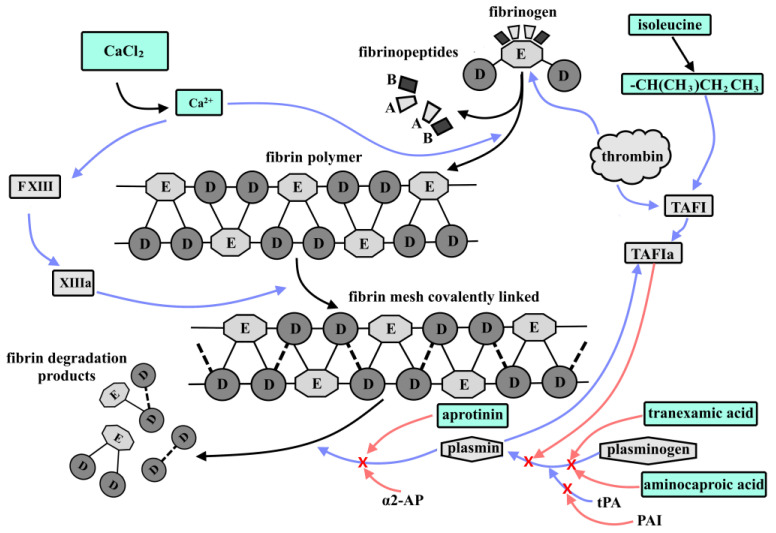
Schematic diagram of the interactions between fibrinolysis activators and inhibitors in the fibrin glue model. As a result of the activation of the coagulation cascade, under the influence of thrombin, 2 fibrinopeptides A and B are cleaved from fibrinogen, thereby forming fibrin monomer. Subsequently, fibrin monomers, under the influence of Ca^2+^ ions, which can give the connection additional strength, spontaneously assemble in a semi-step manner, forming fibrin polymer. After this, under the influence of XIIIa, covalent cross-linking of D-domains occurs, thereby forming a fibrin mesh. Fibrinolysis is regulated by plasminogen, which, under the influence of tPA, is converted into its active form, plasmin. Plasmin, in turn, activates the process of fibrin clot degradation. A number of compounds play a key role in inhibiting fibrinolysis: (1) tPA activity is inhibited by plasminogen activator inhibitors 1 and 2 (PAI). Plasmin itself can be directly inhibited by α2-AP and aprotinin; (2) isoleucine residues stabilize TAFI, after which TAFI, under the action of trypsin-like proteases—thrombin and plasmin, is converted into its active form—TAFIa, which inhibits the reaction of plasmin formation from plasminogen, thereby preventing lysis of the fibrin clot; (3) tranexamic acid and aminocaproic acid, similar to the action of TAFI, prevent the formation of plasmin from plasminogen by inhibiting the lysis of the fibrin mesh. Blue arrows indicate activation processes, while red arrows with red crosses at the ends indicate inhibition processes. Green color indicates compounds added to fibrin glue for additional clot stabilization and inhibition of fibrin mesh lysis.

To reduce the rate of fibrin lysis of the FG fibrinogen component, various substances with antifibrinolytic properties, such as aprotinin, aminocaproic acid, or tranexamic acid, can be added (Figure 3). Siedentop K. et al. assessed the stability of fibrin glue against fibrinolysis after adding aprotinin and aminocaproic acid. According to these researchers, fibrin glue containing aprotinin could still be detected in rat tissue over a period of 6 days, whereas the effect of aminocaproic acid lasted only for 24 h, with residual fragments found on Days 2 and 6 [85]. However, Marx G. and Mou X. demonstrated that the addition of aprotinin to fibrin glue did slow down fibrinolysis, but it also contributed to a slower formation of granulation tissue [86]. Tranexamic acid is a synthetic analog of lysine that competitively inhibits plasminogen conversion to plasmin, resulting in a decrease in the clot fibrinolysis reaction [87]. Such properties of tranexamic acid mean that it is a component that is often added to fibrin glues to increase the lifetime of the fibrin clot and improve its physicochemical properties, namely its stability and strength [88]. However, some studies have demonstrated a negative effect of tranexamic acid on cells [78].

Further modifications for specific applications can be performed by adding various biologically active substances to fibrin glues. It is known that the fibrin matrix can be used as a carrier of protein factors, ensuring their prolonged release [79,80]. In the light of the latter, research is now aimed at modifying fibrin glues by enriching them with growth factors to increase their regenerative potential. For example, FGs have been supplemented with vascular endothelial growth factor (VEGF), transforming growth factor-B1 (TGF-β1), basic fibroblast growth factor (bFGF), and with growth factor-binding proteins such as insulin-like growth factor (IGF)-binding protein-3 [81,89].

A promising FG modification is one that allows for localized prevention [90] of infections that can potentially accompany any surgical intervention [91,92]. To ensure antibacterial activity, antibiotics can be introduced into the fibrin glue [93,94]. In vitro studies of such antibacterial activity have demonstrated that fibrin glues in combination with antibiotics such as vancomycin, ciprofloxacin, teicoplanin, cefoxitin, and gentamicin can have a pronounced antibacterial effect on such pathogens as *Staphylococcus aureus.* Antibiotics contained in fibrin glue clots have been shown to result in the continuous diffusion of medications into the environment over 5–7 days, effectively inhibiting bacterial growth [95]. Animal studies have demonstrated that supplemented fibrin glue provides prolonged release of vancomycin, effectively suppressing local infection [96]. While the previous studies focused on preserving the antibacterial activity of the medications themselves, Thompson D.F. and Davis T.W. primarily paid attention to their effect on the mechanical properties of the fibrin matrix. For example, cefotaxime and gentamicin were found to extend clotting time and potentially to reduce the adhesion strength of the fibrin matrix. This change might impact the efficacy of fibrin glue as a tissue adhesive in clinical settings [97]. Several overviews have summarized the experience of studying fibrin glue as a delivery system for antimicrobial agents. It has been shown that the inclusion of antibiotics in fibrin glues promotes their effective, site-targeted, and long-lasting antimicrobial activity [98,99].

At the same time, it should be noted that, although the introduction of such components as antifibrinolytics, growth factors, and antibiotics into fibrin glues has been studied, so far they have not been widely used in commercial products.

## 5. Fibrin Glues—Experimental Studies

The use of FG is not limited to its hemostatic function; its clinical potential is significantly broader, as evidenced by experimental in vitro and animal model studies. A number of in vitro studies have been devoted to the assessment of fibrin glue as a delivery system. For example, Yu W. et al. (2023) studied fibrin glue as a delivery system for the secretome of mesenchymal stem cells [100]. This in vitro model demonstrated the controlled release of growth factors (FGF-2, VEGF, and IGF-1) over 10 days. Another in vitro model showed the use of fibrin glue as a delivery system for recombinant bone morphogenetic protein for tissue engineering. The authors confirmed localized, controlled protein release over 10 days. Moreover, a similar comparative study found that with rhBMP9 contained in fibrin glue, rather than in a standard commercial glue, alkaline phosphatase activity significantly increased by Day 7, and the mRNA levels of the osteoblast differentiation markers, bone sialoprotein and osteocalcin, also grew by Day 14 [101].

Animal studies with fibrin glues have been conducted to expand their scope of application and to develop new treatment techniques. For example, Abdelkader A. (2016) demonstrated in an animal model (rabbits) that fibrin glue can act as a safe and effective replacement for sutures during ophthalmological surgeries, improving corneal flap edge healing and increasing the resultant mechanical strength [102].

Erica Akhter T. et al. (2024) demonstrated the use of fibrin glue to fix the proximal and distal stumps of injured sciatic nerves in rats [103]. This technique allowed the successful alignment of the nerve stumps, which facilitated complete nerve remodeling, and axon re-innervation of the muscles of the lower hind limb. Zabbia G. et al. (2024) also studied sciatic nerve regeneration in a rat model [104]. The animals were divided into two groups: a control group, where standard neurorrhaphy was used for nerve fusion using an autograft with inverted polarity, and an experimental group, where the autograft was fixed with fibrin glue. It was shown that there were no significant differences between the outcomes for the groups. At the same time, a relative improvement in the number of motor units of the anterior tibial muscle was seen in the experimental group. Although the use of fibrin glue to fix sciatic nerve stumps provides no outstanding results in terms of functional recovery, the efficiency of fixation is comparable to that of standard neurorrhaphy. Thus, this simpler and faster technique for fixing nerve stumps using fibrin glue may deserve wider clinical application.

Mayrhofer-Schmid M. et al. (2024) assessed the potential of fibrin glue to reduce scar tissue formation around peripheral nerves, specifically the sciatic nerve in rats [105]. The experimental group, where the injured sciatic nerve was fixed with fibrin glue, demonstrated a reduced functional recovery time (6 weeks vs. 9 weeks) compared to the group of rats where no fibrin glue was used. The experimental group also demonstrated reduced perineural scar tissue formation and greater fiber density, myelin thickness, axonal thickness, and myelinated fiber thickness compared to the control group. Thus the results of the study show that fixing of the injured nerve with fibrin glue significantly reduces scar tissue formation and improves nerve regeneration, both at the microscopic and functional levels.

A study of the feasibility of the endoscopic application of fibrin glue to seal experimentally induced post-intubation tracheal lacerations showed that endoscopically delivered fibrin was a suitable technique for sealing such injuries [106]. This study was conducted on cat cadavers.

A study of the effect of fibrin glue on the regeneration of tibial bone defects in rats found that it stimulated bone growth and increased tibial density and bone mineral content. The results showed an increase in bone volume, with the formation of a high number of trabeculae of greater thickness in the groups using either fibrin glue or fibrin glue in combination with autografts [107].

Dejyong K. et al. (2017) assessed the effectiveness of fibrin glue in skin engraftment in pigs [108]. Their study demonstrated that the advantage of its use was in decreasing skin graft rejection rates by approximately 31.3–37.5% compared to the control group, as well as in its stimulation of angiogenesis. Fibrin glue has also been demonstrated to be effective in healing colorectal anastomosis in a rat model of diabetes mellitus. The glue was used to seal the anastomosis in both normoglycemic control rats and in severely hyperglycemic rats. Pathological examination revealed improved tissue remodeling in both groups, with a significant decrease in inflammation and an increase in the collagen and fibroblast content [109]. In another study, fibrin glue was used to seal localized colon perforations in a rat model. This study demonstrated the efficacy and safety of the fibrin glue for sealing and healing laparoscopic bowel injuries less than 2 mm in size. No recurrence of bowel perforations was seen. Histopathological results revealed a decrease in the inflammatory response, reduced fibrosis, and improved tissue regeneration compared to the control group that received standard suturing [110].

Luckanahasaporn S. et al. (2019) demonstrated the hemostatic efficacy of fibrin glue in liver biopsies in pigs [111]. Whole blood clotting time, bleeding time, and the number of hemorrhages were significantly lower in the fibrin glue group compared to the control group.

Nikolaou C. et al. (2024) studied the efficacy of fibrin glue in burn treatments in a rat model [112]. The results showed that the fibrin glue group had higher collagen expression compared to both the control group and to a silver sulfadiazine-treated group. Histopathological analysis demonstrated pronounced leukocyte infiltration, high neovascularization, and high fibroblast expression in the glue group compared to the other two groups. Thus, successful in vitro and in vivo studies have provided the basis for the appropriateness of the widespread clinical use of FGs, as well as continuing to expand the scope of their application.

## 6. Fibrin Glues in Clinical Practice

**Figure 4:**
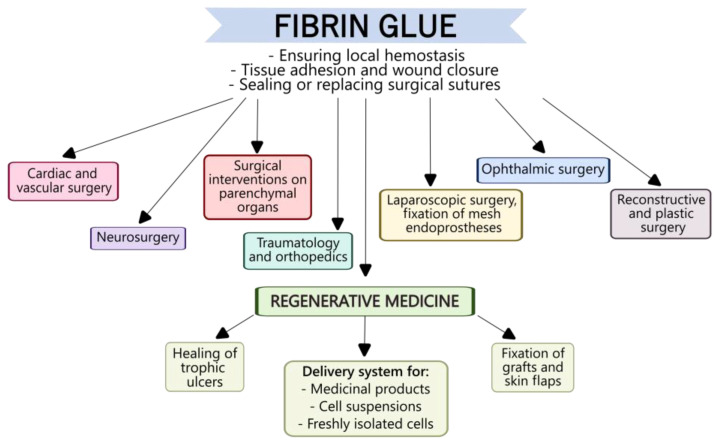
Use of fibrin glue in clinical practice.

Currently, fibrin glues have shown themselves to be valuable in various surgical interventions, leading to their widespread use in clinical practice (Figure 4). The primary purpose of using fibrin glues is to stop bleeding during surgery and hermetically to seal wound surfaces of various organs and tissues, either in addition to or instead of using surgical sutures.

Cardiac surgery was one of the first areas of medicine to use fibrin sealants. The most common use of fibrin glues in cardiac and vascular surgery is to seal suture lines. It was shown that fibrin sealants, in combination with external sutures, can ensure effective hemostatic control in various clinical cases, such as in patients with congenital heart defects [113], as well as the successful healing of cardiac ruptures due to gunshot wounds. Moreover, the use of fibrin sealants typically does not cause any complications either during surgery or in the postoperative period. In peripheral vascular surgery, VISTASEAL™ and Evicel^®^ have demonstrated better results than manual compression approaches [114].

Fibrin sealants have proved themselves in neurosurgery [115,116]. The use of fibrin glues has been shown to facilitate nerve tissue regeneration in a manner comparable to sutures. In this respect, Koopman J.E. et al. believe that the combined use of sutures and fibrin sealants is even more effective for nerve fiber regeneration [117]. The use of fibrin sealants as carriers for mesenchymal stromal cells (MSCs) has been suggested as a less invasive strategy for stimulating nerve tissue regeneration. The combined use of fibrin sealants and MSCs has great potential for repairing damage to both the nervous system and the surrounding tissues; however, the clinical efficacy of this treatment requires further study [118].

Parenchymal bleeding is one of the most difficult types of bleeding to control. For example, even a small incision in the surface of the liver or spleen provokes excessive bleeding that is difficult to control using traditional techniques. The application of deep sutures can result in additional damage, while electrocoagulation provokes local tissue necrosis. This leads to complications both during surgery and in the postoperative period. In such situations, fibrin glue, independently or as an ad hoc agent, can be an effective means of controlling the hemostasis. For example, Hwang S. et al. (2021) described a clinical case in which fibrin glue was used in combination with hepatorrhaphy to stop excessive postoperative bleeding from the surface of a liver incision [119]. The authors noted that the combination of FG with superficial sutures effectively restored hemostasis, despite the patient taking anticoagulants before and during the postoperative period. The patient’s condition was monitored for six months, and throughout the follow-up no complications were observed. A number of studies have demonstrated the positive effect of FGs in thyroid surgery. Reductions in the total volume of drainage, in operative times, and in lengths of hospital stay have been recorded for such interventions [120,121].

As most commercial fibrin glues are intended for use on dry surfaces, there have been concerns that their application directly onto a wet, bleeding surface may not be sufficiently effective. In this respect, Hwang S. et al. proposed injecting FG into the parenchyma, to a depth of 0.5–1 cm from the bleeding surface, and then slowly withdrawing the needle with continuous administration of the FG. The authors noted that, using such an application technique, droplets of the FG were distributed throughout the parenchyma, causing both mechanical compression of the small, bleeding vessels and immediate hemostasis on the liver surface. The authors tested the described technique in eight cases of liver transplantation and resection; in all patients, immediate and complete cessation of bleeding was observed [122]. This result gives us confidence in suggesting that FGs can be effectively used as independent hemostatic agents in such clinical situations. It has thus been demonstrated that the use of FG provides the most effective means of hemostatic control during liver resection [123].

Fibrin glues can also be applicable in cases other than major surgical interventions. For example, splenic puncture biopsy is an important diagnostic procedure that can relieve patients of the need for major surgery and splenectomy. However, this procedure is associated with a high risk of severe bleeding due to the copious blood flow to the spleen. Segger L. et al. proposed a technique for closing the puncture channel during splenic biopsy using TISSEL/TISSUCOL fibrin glue as a sealant. The efficacy and safety of this technique were confirmed in 20 patients, but the authors noted the need for a larger study [124].

Given that the developing fibrin matrix can act as a scaffold to settle cells recruited from surrounding tissues in a manner similar to that of a natural blood clot, FGs can promote tissue regeneration. Thus, fibrin sealants are quite widely used to treat wounds of various etiologies, such as chronically non-healing ulcers, especially of venous origin, that are usually very difficult to treat with traditional means. In the treatment of peptic ulcers, fibrin sealants, autologous and allogeneic, have proven themselves effective both as graft fixators and as keratinocyte delivery systems promoting faster healing of a range of types of such ulcers. In the treatment of burns, the traditional means of treatment is skin graft transplantation. The use of FGs rather than staples [125] to fix skin flaps reduces the duration of surgery, increases the rate of graft incorporation, and significantly decreases pain in patients over the postoperative period [126]. These benefits justify the higher cost of FGs compared to the use of staples [127]. In the treatment of extensive skin wounds, when autologous material is insufficient to close the wound, FGs can be used as carriers for cell suspensions. For instance, it has been shown that the application of keratinocytes or freshly isolated skin cells in a fibrin sealant results in a uniform distribution of the cells over the wound surface without causing any damage to the cells [128,129]. Consequently, FGs are being used in the current development of new technologies and strategies for the treatment of skin wound defects.

Fibrin sealants are also applied in traumatology and orthopedics [130]. For example, FGs can reduce either the need for blood transfusions or the volume of transfused material required during hip hemiarthroplasty, confirming their effectiveness as a means of hemostatic control [131]. However, the use of FGs as gluing agents in orthopedic surgeries does require further research and development. For instance, in the case of bone tissue restoration, the use of FGs is limited to fractures that are not subsequently subjected to mechanical force at the site of application. This is necessary due to the relatively low adhesion strength to bone fragments, despite good fixation [132]. Commercially available FGs show good adhesion properties in cartilage tissue restoration but are still insufficient, alone, to retain cartilage implants [133]. Thus, to ensure the reliable fixing of bone and cartilage implants, FG modification is required. This can be achieved by introducing additional components into the FGs. For example, a commercial DeNovo NT allograft has been developed (ISTO St. Louis, MO, USA; distributed by Zimmer, Warsaw, IN, USA); it includes crushed allogeneic human cartilage fragments to be secured within the fibrin glue. The published results of clinical studies have proven the efficacy of this approach in articular cartilage restoration [134]. Another application of fibrin glue in traumatology and orthopedics is the use of fibrin sealant in combination with mesenchymal stem cells (MSCs). Several preclinical studies have demonstrated the use of this fibrin glue composite in the treatment of defects of the cranial vault, femur, and maxilla and mandible. Clinical studies have also described its use in the treatment of osteoarthritis and cartilage damage in the knee joint [16].

Fibrin glues are also actively being used in the field of maxillofacial surgery and as alternatives for the fixation of bone grafts. For example, in a study by Ozkul E. et al. (2025), fibrin glue was successfully used to fix an iliac bone autograft in the treatment of an orbital floor fracture [135]. In the work of Um J.H. et al. (2022), a comparative study was conducted into the fixation of isolated fractures of the anterior wall of the maxilla using either absorbable mesh or fibrin glue [136]. The group treated using only fibrin glue demonstrated better results in terms of length of hospital stay and the incidence of acute complications. In addition, fibrin glues can be used to fix cartilage grafts in rhinoplasty [137].

In ophthalmic surgery, the search for an alternative to surgical sutures is of particular relevance. Here, too, the use of fibrin glues has provided an effective and safe alternative to traditional suture materials. The wide range of commercially available fibrin sealants allows surgeons to choose the most appropriate tool—for example, a material with slower polymerisation for easier tissue manipulation after fibrin application, or, vice versa, one with a higher polymerisation rate to reduce surgical duration [138]. Moreover, Bouhout S. et al. (2022) in their recent study also demonstrated the cost-effectiveness of using fibrin sealants compared to sutures [139]. Despite their higher material cost, fibrin sealants significantly reduce surgical time, thereby reducing overall costs.

In reconstructive plastic surgery, minimal procedural damage and the prevention of complications are crucial during surgical interventions. In this field, fibrin sealants have proved themselves effective as means of hemostatic control, ensuring avoidance of postoperative complications and the need for active drainage [140,141]. It has been shown that the use of FGs can reduce the number of seromas and hematomas during the postoperative period, as well as significantly decreasing the time subsequently required for drainage [142]. In transconjunctival blepharoplasty, the use of fibrin glue significantly reduced both the severity of skin discoloration and of hematomas compared to suturing [143]. Retrospective analysis of patients who underwent external rhinoplasty with the use of FGs during surgery has shown positive dynamics over the long term, while there have been no adverse effects on the aesthetic results [144].

In bariatric surgery, the use of fibrin sealants along suture lines has been demonstrated to offer a favorable trend in reducing the incidence of postoperative complications [145]. However, according to Chen Y.S. et al., the effectiveness of FG in such a reduction was not significantly different from that of the use of dressings, although the application of FG decreased the intervention time [146].

Fibrin sealants are highly recommended in laparoscopic surgery, where they provide an alternative to sutures and staples. Several studies have demonstrated that in hernioplasty, mesh fixing with FG during a laparoscopic approach was well up to the standard of classical techniques [147,148]. Moreover, a decrease in the incidence of postoperative pain and of hematomas in patients when using FG was noted [149].

By contrast, specialists in periodontal surgery consider the use of FGs inappropriate as an alternative to surgical sutures. The reason for this is the insufficiency of the evidence base for the clinical use of FGs without the application of sutures. The authors note the need for additional clinical trials involving faithful adherence to protocols [150].

Thus, in general, the use of FGs successfully solves the issue of hemostasis during surgery and enables the sealing of wound surfaces of various organs and tissues, particularly in difficult-to-reach areas where the use of surgical sutures is problematic. Moreover, FGs can actively limit the extent of inflammatory processes in the suture area, reducing the risk of complications and accelerating regenerative activity. All these considerations have contributed to the widespread use of FGs across various surgical fields.

## 7. Conclusions

Fibrin glues exhibit good biocompatibility and adhesive and hemostatic properties, making them excellent for use in surgery, traumatology, dentistry, regenerative medicine, and other fields. The most important advantages of fibrin glues are their rapid resorption and lack of toxicity. However, fibrin glues do have disadvantages, including their high production costs, the limited lifetime of the fibrin clots, and—in some cases—the need to consider the individual characteristics of the patient.

Fibrin glues are promising products with a wide range of applications in various areas. However, some aspects of their production and use require further study. For example, the long-term use of fibrin glues and potential optimization of their compositions are still being discussed.

Currently, fibrin glues are not only important tools for solving clinical issues but also innovative platforms that open up new horizons for research into drug delivery and combination therapies. Study of the interaction of such glues with cells and tissues will help to create a new generation of fibrin glues with adjustable properties.

## Data Availability

No new data were created or analyzed in this study. Data sharing is not applicable to this article.

## Abbreviations

The following abbreviations are used in this manuscript:

FG	Fibrin glue
FS	Fibrin sealant
FA	Fibrin adhesive
BAS	Biologically active substances
TAFI	Thrombin-activatable fibrinolysis inhibitor
